# The effects of creep feed composition and form and nursery diet complexity on small intestinal morphology and jejunal mucosa-specific enzyme activities after weaning in pigs

**DOI:** 10.1093/jas/skac138

**Published:** 2022-04-15

**Authors:** Brenda Christensen, Lee-Anne Huber

**Affiliations:** Department of Animal Biosciences, University of Guelph, Guelph, ON N1G 2W1, Canada

**Keywords:** creep feed, enzyme activity, histomorphology, milk replacer, nursery diet complexity

## Abstract

Fifty-six litters from first-parity sows standardized to 12 piglets were used to determine the effects of creep feed composition and form and the provision of low- or high-complexity nursery diets on the evolution of small intestinal histomorphology and jejunal mucosa-specific enzyme activities postweaning. At 5 d of age, litters (initial bodyweight [BW] 2.31 ± 0.61 kg) were assigned to one of four creep feeding regimens (*n* = 14): 1) commercial creep feed (**COM**), 2) liquid milk replacer (**LMR**), 3) pelleted milk replacer (**PMR**), or 4) no creep feed (**NO**). At weaning (21 d of age), six pigs per litter were provided a **HIGH**- (contained highly digestible animal proteins) or **LOW**- (contained corn and soybean meal as main protein sources) complexity nursery diet (*n* = 7). At 21, 28, and 59 d of age, two pigs per pen (one castrated male and one female) were euthanized, and ileal and jejunal segments for histomorphological measurements and jejunal mucosal scrapings were collected to determine specific mucosa enzyme activities. At weaning, pigs provided COM had a greater ileal absorptive capacity (**M**) than LMR or NO, which were not different (14.1 vs. 10.4 and 10.5 ± 0.9 μm^2^; *P* < 0.05); PMR was intermediate. On days 28 and 59, M was not different among pigs regardless of creep feed treatments. Pigs fed LOW had reduced jejunal villus height (**VH**; *P <* 0.001) and M (*P <* 0.001) on day 28 vs. day 21. The VH and M were not different for pigs fed HIGH or LOW by the end of the nursery period. For all dietary treatments except COM-HIGH and COM-LOW, jejunal mucosal maltase-specific activity was not different between days 21 and 28 of age but greater on day 59 (*P* < 0.05). For pigs that received COM-HIGH, maltase-specific activity was not different between days 21 and 28 but greater on day 59 than day 28 (*P* < 0.05). For pigs that received COM-LOW, maltase-specific activity was not different between days 21, 28, and 59. Regardless of creep or nursery treatment, sucrase-specific activity was the greatest on day 59, followed by days 21 and 28 (*P <* 0.001), and lactase-specific activity was greater on day 21 than on days 28 and 59 (*P <* 0.001), which were not different. Therefore, pigs that provided LOW diet had greater villus atrophy and reduced M during the first week after weaning vs. pigs that provided HIGH, regardless of creep feeding regimen, but were able to recover by the end of the nursery period.

## Lay Summary

After weaning, the piglet digestive tract must adapt in order to effectively break down and absorb nutrients derived from plant-based ingredients, contributing to the postweaning growth lag. Feeding strategies applied both preweaning and postweaning with the intent to assist in intestinal adaptation have been developed; however, little work has been done examining the interaction between creep and nursery feeding strategies on intestinal histomorphology and jejunal mucosa-specific enzyme activities after weaning. In the current study, piglets that provided creep feed with higher starch content had greater jejunal mucosa maltase-specific enzyme activity and ileal absorptive capacity at weaning. However, these parameters were still negatively affected by weaning, regardless of nursery feeding strategy. Additionally, nursery diets with greater inclusion of soybean meal to replace animal protein sources (low complexity) exacerbated reductions in jejunal absorptive capacity. Conversely, by the end of the nursery period, there were no differences in intestinal histomorphology and jejunal mucosa-specific enzyme activities, regardless of creep or nursery feeding program. Therefore, exposing piglets to starch during creep feeding and reducing the inclusion of soybean meal in nursery diets improved intestinal morphology and mucosa disaccharidase activity directly following weaning, but benefits did not extend to the end of the nursery period.

## Teaser

Foregoing creep feed and feeding low-complexity nursery diets to pigs did not have lasting negative effects on gastrointestinal physiology.

## Introduction

During the suckling phase, milk production by the sow limits piglet growth, which is especially evident for first-parity sows and sows with large litters (Strathe et al., 2017). Additionally, after weaning, piglets experience a growth lag due to stressors including the abrupt change in diet composition and form (milk vs. cereal grains; liquid vs. pelleted) and the accompanying reduction in feed intake (Pluske et al., 2007; Sulabo et al., 2010; Muns and Magowan, 2018). Conversely, piglets that consume creep feed or additional supplemental milk replacer during the suckling phase typically have a shorter fasting interval and increased feed intake after weaning, resulting in improvements in growth compared with those not provided creep feed (Bruininx et al., 2002; Miller et al., 2012). Moreover, despite some piglets sampling the sows’ diet during the suckling period (Wattanakul et al., 2005), most piglets that are not offered creep feed have no exposure to plant-based ingredients and the accompanying adaptations to the digestive tract necessary to effectively process plant-derived components (Koo et al., 2017). For example, the adaptation of pancreatic and brush border enzyme activities occurs during the first 2 wk after weaning with an initial reduction in activity, after which the activities surpass preweaning levels (Hampson and Kidder, 1986; Jensen et al., 1997; Levesque et al., 2012). This lag in digestive enzyme activity adaption is related to the postweaning growth lag, with the greatest reduction in growth coinciding with the lowest enzyme activities (Hampson and Kidder, 1986).

Nursery diets are typically formulated to be highly digestible by the immature gastrointestinal tract of the piglet by using animal-derived ingredients (e.g., lactose, whey, and fishmeal; Ma et al., 2019). Alternatively, soybean meal is a less expensive protein source but is less digestible (Cervantes-Pahm and Stein, 2010) and contains antigenic compounds that can lead to additional intestinal inflammation (Koo et al., 2017, 2020; Ma et al., 2019). Pigs fed corn- and soybean meal-based (low complexity) diets immediately after weaning experience an initial reduction in average daily gain but are ultimately able to achieve BW comparable to pigs fed highly digestible nursery diets (high complexity) via compensatory growth (Skinner et al., 2014; Huber et al., 2018). The hypothesis was that the provision of COM would assist in the maturation of the intestinal tract (i.e., specific mucosal enzyme activities and morphology) preweaning, which would expedite adaptation to low-complexity nursery diets. The objective of this study was to determine the effect of creep feed composition and form and nursery diet complexity on the evolution of small intestinal morphology and jejunal mucosa-specific enzyme activities after weaning.

## Materials and Methods

### Animals, dietary treatments, and feeding

The experimental protocol was approved by the University of Guelph Animal Care Committee and followed Canadian Council on Animal Care guidelines (CCAC, 2009; AUP #4044). The study was conducted at the University of Guelph Arkell Swine Research Station (Guelph, ON, Canada).

Six hundred seventy-two piglets from first-parity sows were recruited over seven breeding batches (blocks). All litters were standardized to 12 piglets by 48 h following parturition. On 5 ± 0.3 d of age (initial BW 2.38 ± 0.02 kg), litters were assigned to one of four creep feeding regimens: 1) commercial creep feed with corn and fishmeal as major ingredients (**COM**; Floradale Feedmill Ltd., Floradale, ON, Canada), 2) liquid milk replacer (**LMR**), 3) pelleted milk replacer (**PMR**), or 4) no creep feed (**NO**; *n* = 14). The LMR (powder) and PMR had similar ingredient composition; corn was added to the PMR to assist with pelleting. The LMR (powder) and PMR had matched levels of net energy, crude protein, crude fat, and standardized ileal digestible lysine (Grober Nutrition, Cambridge, ON, Canada; Christensen and Huber, 2021). All creep feeds also contained 1% (wt/wt) brilliant blue dye to visually identify individual piglets as creep feed consumers via the appearance in feces, to assist in the selection of pigs at weaning. At weaning (21 ± 2.1 d of age), six median BW piglets per litter (three castrated males and three females) that had at least two blue fecal swabs among days 15, 17, and 21 of age (apart from NO) were placed in a nursery pen (one litter per pen); only pigs that were identified as creep feed consumers were used for tissue collection at weaning and for the nursery portion of the study. During the nursery period, litters were provided either a **HIGH**- or a **LOW**-complexity diet in a three-phase feeding program. Phase I was fed for 7 d, phase II for 14 d, and phase III for 17 d (Christensen and Huber, 2021).

### Experimental procedures

At weaning (21 d of age) and 1 week (28 d of age) and 38 d after weaning (59 d of age), 2 pigs per litter were randomly selected (1 castrated male and 1 female; 14 pigs per treatment) and were euthanized with an intra-cardiac injection of 3 mL of Euthasol (Virbac, TX; 21 and 28 d of age) or by electrical stunning followed by exsanguination (59 d of age). Immediately thereafter, the entire gastrointestinal tract was excised, and intestinal samples were collected. Mucosal scrapings from the center of the jejunum (20 cm) were collected by using a glass slide to separate mucosa from connective tissue. Mucosal samples were flash-frozen in liquid nitrogen and stored at −80 °C until further analysis. An additional 5-cm segment of the jejunum (approximately 1.5 m distal to the ligament of Trietz) and ileum (approximately 0.5 m proximal to the ileo-cecal junction) was collected, rinsed with physiological saline (0.9% NaCl), and stored in 10% formalin until further analysis. Jejunal and ileal tissue segments were prepared for histology analysis according to the procedures of Carleton et al. (1980). Measurements of villus height (**VH**), villus width (**VW**), crypt depth (**CD**), and crypt width (**CW**) were collected from 10 villi in each intestinal segment (Leica microsystems Inc., Wetzlar, Germany; and Openlab Computer Imaging System, Perkin Elmer, Waltham, MA). The VH:CD and absorptive capacity (**M**) were calculated using the average values for each VH, VW, and CW for each segment per pig. Absorptive capacity (equation 1) was calculated according to Kisielinski et al. (2002):


$$\begin{array}{l} M=\frac{\left(VW\cdot VH\right)+{\left(\frac{VW}{2}+\frac{CW}{2}\right)}^{2}-{\left(\frac{VW}{2}\right)}^{2}}{{\left(\frac{VW}{2}+\frac{CW}{2}\right)}^{2}} \end{array}$$ (1)

### Determination of specific enzyme activity

Mucosal samples were homogenized (1:20; wt/v) using PowerGen 125 homogenizer (Fisher Scientific, Toronto, ON) in homogenization buffer (50 mM d-mannitol, 10 mM Trizma∙HCl, and 10 mM Hepes diluted in Milli-Q water and adjusted to pH of 7.4 using 2.0 M NaOH), aliquoted into microcentrifuge tubes, and stored at −80 °C until further analysis. Mucosal homogenates were analyzed for protein concentration in duplicate according to manufacturer instructions (Bio-Rad, Hercules, CA); bovine serum albumin (Sigma Chemical Company, St. Louise, MO) was used as the protein standard. Specific enzyme activities for sucrase (sucrose-isomaltase, E.C. 3.2.1.48), maltase (maltase-glucoamylase, E.C. 3.2.1.20), and lactase (lactase-phlorizin hydrolase, E.C. 3.2.1.108) were conducted according to Dahlqvist (1968). The reaction was stopped with BaOH, and ZnSO_4_ was used to precipitate the protein. Sucrase-, maltase-, and lactase-specific activities were determined at 37 °C for 10 min in a final volume of 200 μL using glucose oxidase as the reagent (Point Scientific Inc., Canton, MI) at substrate levels of 312.5, 75, and 312.5 mM, respectively. A lactase standard that was prepared in the same way as the samples was included on each plate and used to calculate a correction factor (CF_Lactase_; equation 2) to adjust optical densities for individual plates. The optical density was measured at 500 nm with a reference wavelength of 650 nm. The amount of enzyme necessary to hydrolyze 1 nmol of the respective substrate per minute at 37 °C per mg of protein at a pH of 6.0 was defined as one unit:


$$\begin{array}{l} CF_{Lactase}=\;\frac{Overall\;mean\;of\;absorbance\;of\;lactase\;standard}{Absorbance\;of\;lactase\;standard\;on\;individual\;plate} \end{array}$$ (2)

### Statistical analysis

The statistical analyses for histomorphological measurements and specific enzyme activities were conducted using the GLIMMIX procedure of SAS (University Edition; SAS Ins. Inc., Cary, NC) with creep feed treatment, nursery diet treatment, and time, and the interactions between creep feed treatment, nursery diet treatment, and time as the main effects and block as a random effect. In all analyses, the degrees of freedom were calculated with Kenward–Roger’s adjustment for repeated measures, and outliers were detected using the univariate procedure. Model residuals were assessed using scatter and box plot of studentized residuals for homogeneity of variance and Q–Q plot and Shapiro–Wilk test for normal distribution. Mean comparisons were conducted using Tukey–Kramer post hoc test to separate means. Probability (***P***) values of less than 0.05 were considered significant.

## Results

### Small intestinal histomorphology

Jejunal histomorphology measurements were not influenced by the three-way interaction between creep treatment, nursery treatment, and time; by the two-way interactions between creep treatment and time or between creep and nursery treatments; or by the main effects of creep and nursery treatment (data not shown). Jejunal VH and M were influenced by the interaction between nursery treatment and time (*P* < 0.05; Table 1). Jejunal VH and M were reduced between 21 and 28 d of age for pigs that received LOW (*P* < 0.05) but were not different between 21 and 28 d of age for pigs that received HIGH. Jejunal VH was less on days 21 and 28 compared with day 59 (*P* < 0.05) and not different between nursery treatments within day. Likewise, M was greater on days 21 and 59 compared with M on day 28 (*P* < 0.05) and was not different between nursery treatments within day. The VW, CD, CW, and VH:CD were not influenced by the interaction between nursery treatment and time but were influenced by the main effect of time (Table 1). The VW and CD were not different between days 21 and 28 of age and less than on day 59 (*P* < 0.05). The CW was less on day 21 of age than on days 28 and 59 (*P* < 0.05), which were not different. The VH:CD was greater on days 21 and 59 of age than on day 28 (*P* < 0.05) and greater on day 21 than on day 59 (*P* < 0.05).

**Table 1.** Effect of nursery treatment and time after weaning on jejunal and ileal histomorphology

	Day 21 of age^1^		Day 28 of age		Day 59 of age		SEM^3^	*P*-values^2^		
	HIGH	LOW	HIGH	LOW	HIGH	LOW		Nursery	Time	Nursery * Time
No.^4^	28	28	28	28	28	28				
Jejunum										
Villus height (VH), μm	475^bc^	534^b^	416^cd^	376^d^	615^a^	663^a^	32	0.156	<0.001	0.018
Villus width, μm	109	108	100	107	133	132	5	0.660	<0.001	0.587
Crypt depth (CD), μm	44	38	45	43	49	50	2	0.134	<0.001	0.188
Crypt width, μm	169	177	259	277	275	263	13	0.624	<0.001	0.373
Absorptive capacity, μm^2^	10.1^ab^	11.2^a^	8.6^bc^	7.7^c^	10.5^a^	11.4^a^	0.4	0.233	<0.001	0.018
VH:CD	3.2	3.5	1.6	1.4	2.5	2.7	0.15	0.483	<0.001	0.198
Ileum										
Villus height, μm	539^b^	650^a^	401^c^	374^c^	718^a^	726^a^	39	0.107	<0.001	0.014
Villus width, μm	109	107	109	116	129	123	5	0.911	0.0004	0.331
Crypt depth, μm	38	44	47	48	53	52	4	0.694	0.0041	0.570
Crypt width, μm	169	165	256	283	264	263	14	0.395	<0.001	0.239
Absorptive capacity, μm^2^	11.2	12.5	7.9	7.2	12.4	12.6	0.7	0.489	<0.001	0.097
VH:CD	3.2^ab^	3.9^a^	1.6^c^	1.4^c^	2.9^b^	2.9^b^	0.23	0.331	<0.001	0.030

Pigs were weaned at 21 d of age and received either HIGH- (contained multiple, animal protein sources) or LOW- (contained corn and soybean meal as the main protein sources) complexity nursery diets.

*P*-values for the main effects of nursery treatment, time, and the interaction between nursery treatment and time.

Maximum value for the standard error of the means.

Number of litters evaluated.

Within a row, means without a common superscript differ, *P* < 0.05.

**Figure 1.**
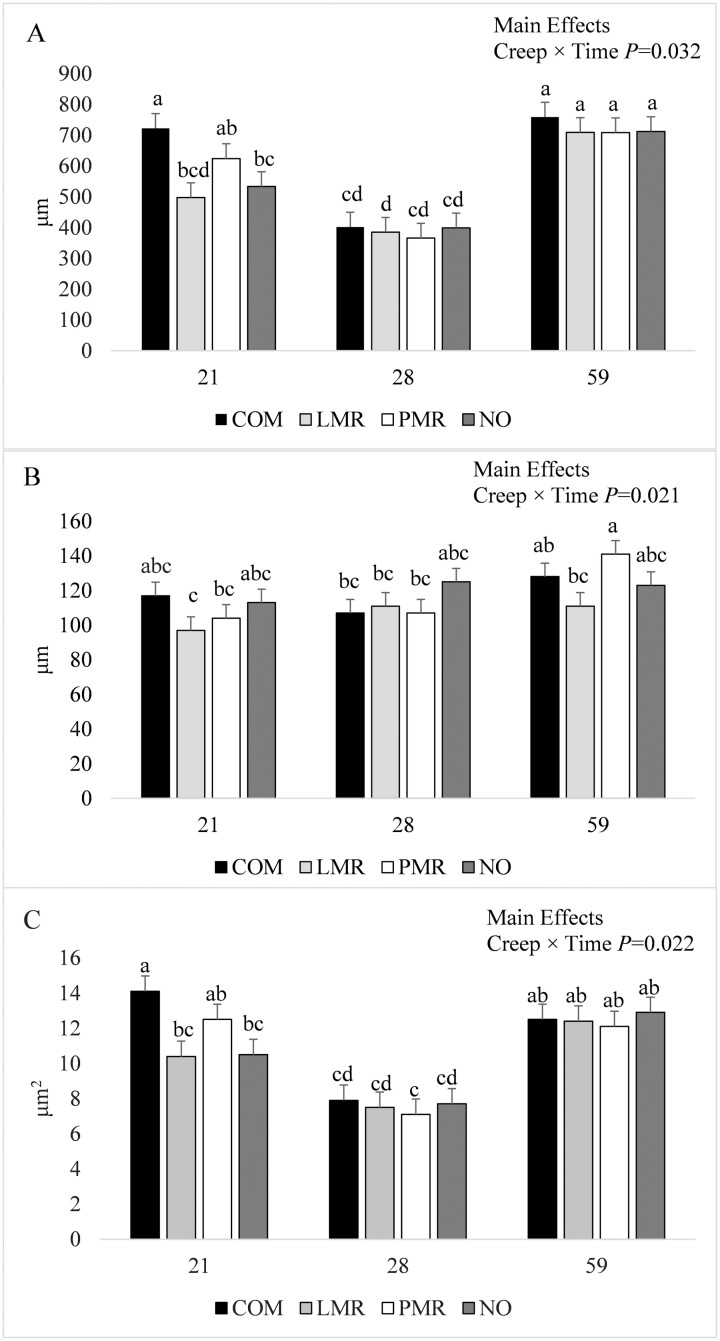
The interaction between creep treatment (COM, commercial, pelleted creep feed; LMP, liquid milk replacer; PMR, pelleted milk replacer; NO, no creep feed offered) and time (days 21, 28, and 59 of age) on ileal villus height (A), villus width (B), and absorptive capacity (C). Values are LSmeans ± SEM, *n* = 14. ^a–d^Means without a common superscript differ; *P* < 0.05.

Ileal histomorphology measurements were not influenced by the interaction between creep and nursery treatments or the main effect of nursery treatment. Ileal VH was not influenced by the interaction between creep treatment, nursery treatment, and time but was influenced by the interaction between creep treatment and time (Figure 1A; *P* < 0.05). On day 21 of age, pigs fed COM had greater VH than those fed LMR or NO creep feed (*P* < 0.05), while intermediate values were observed for PMR. Only pigs fed COM and PMR had lower VH on day 28 vs. day 21 (*P* < 0.05), but on day 59 of age, all pigs had greater VH than on day 28, regardless of creep treatment (*P <* 0.05). The VH was also influenced by the interaction between nursery treatment and time and the main effect of time (*P* < 0.05; Table 1). On day 21 of age, pigs fed LOW had greater VH than pigs fed HIGH (*P* < 0.05), which were both greater than VH on day 28 of age, regardless of nursery treatment (*P* < 0.05). On day 59 of age, VH was greater than for day 28 of age, regardless of nursery treatment (*P* < 0.05), and was greater on day 59 vs. day 21 of age for pigs fed LOW (*P* < 0.05) but was not different between days 59 and 21 for pigs fed HIGH. Regardless of nursery or creep treatments, VH was less on day 28 than days 21 and 59 of age (*P* < 0.05) and greater on day 59 than day 21 of age (*P* < 0.05). Regardless of nursery treatment or time, pigs fed COM had greater VH than those fed NO or LMR (628 vs. 548 and 530 μm; *P <* 0.05; for COM, NO, and LMR, respectively; data not shown), with PMR intermediate.

There was an interaction between creep treatment, nursery treatment, and time for ileal VW (*P* < 0.05; data not shown). The VW was greater on day 59 of age for pigs fed PMR-HIGH compared with pigs fed LMR-HIGH, LMR-LOW, PMR-HIGH, and NO-LOW on day 21 of age and COM-HIGH, COM-LOW, and LMR-HIGH on day 28 of age (*P* < 0.05), with all other creep and nursery treatment combinations intermediate (data not shown). The VW was also influenced by the interaction between creep treatment and time (*P* < 0.05), such that there were no differences among creep treatments on days 21 and 28, but by day 59, only pigs fed PMR had greater VW than on days 21 and 28 (*P* < 0.05), and greater VW than pigs fed LMR (*P* < 0.05; Figure 1B). Regardless of nursery treatment or time, pigs fed NO had greater VW than those fed LMR (120 vs. 106 μm; *P <* 0.05) with PMR and COM intermediate (data not shown). The VW was not influenced by the interaction between nursery treatment and time but was influenced by the main effect of time (*P* < 0.05; Table 1). Regardless of nursery or creep treatments, VW was not different between days 21 and 28 of age, which were both less than VW on day 59 of age (*P* < 0.05).

The CD and CW were not influenced by any interactions or the main effects of creep treatment (data not shown), though both were influenced by the main effect of time (*P* < 0.05; Table 1). Regardless of creep or nursery treatments, the CD was less on day 21 vs. day 59 of age (*P* < 0.05) and intermediate on day 28, and the CW was less on day 21 vs. days 28 and 59 of age (*P* < 0.05), which were not different.

Ileal M was not influenced by the interactions between creep treatment, nursery treatment, and time, or between nursery treatment and time, or by the main effect of creep treatment (data not shown). Ileal M was influenced by the interaction between creep treatment and time (*P* < 0.05; Figure 1C). On day 21 of age, pigs fed COM had greater M than those fed LMR or NO (*P* < 0.05), while intermediate values were observed for pigs fed PMR. Only pigs fed COM and PMR had lower M on day 28 vs. day 21 (*P* < 0.05), but on day 59 of age, all pigs had greater M than on day 28, regardless of creep treatment (*P <* 0.05). Ileal M was influenced by the main effect of time (*P* < 0.05) such that, regardless of creep and nursery treatments, M was less on day 28 than on days 21 and 59 of age (*P* < 0.05), which were not different (Table 1).

The VH:CD in the ileum was not influenced by the interactions between creep treatment and time or between nursery treatment and time or by the main effect of creep treatment (data not shown). The VH:CD was influenced by the interaction between creep treatment, nursery treatment, and time (*P <* 0.01), such that only pigs fed COM-LOW, LMR-HIGH, LMR-LOW, PMR-HIGH, and PMR-LOW had lower VH:CD on day 28 vs. day 21 (*P* < 0.05) and only pigs fed PMR-LOW had greater VH:CD on day 59 vs. day 28 (*P* < 0.05); the VH:CD was not different between days 21, 28, and 59 for pigs fed COM-HIGH, NO-HIGH, and NO-LOW (data not shown). The VH:CD ratio was also influenced by the main effect of time (*P* < 0.05) such that, regardless of creep or nursery treatments, VH:CD was greater on day 21 than on day 59 of age (*P* < 0.05), which were both greater than on day 28 (*P* < 0.05; Table 1).

### Specific jejunal mucosal enzyme activity

**Figure 2.**
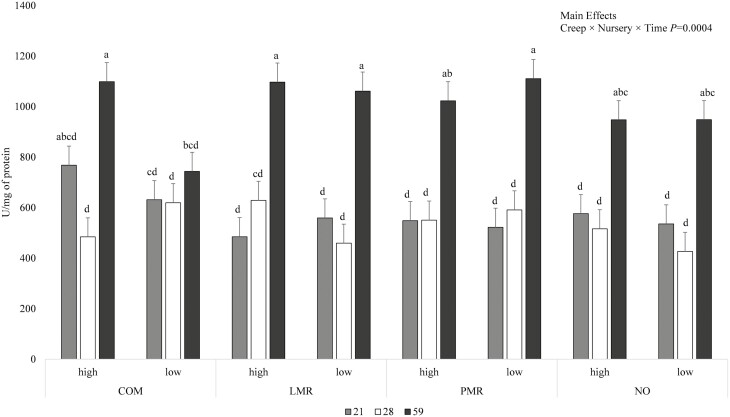
The effects of creep treatment (COM, commercial, pelleted creep feed; LMP, liquid milk replacer; PMR, pelleted milk replacer; NO, no creep feed offered), nursery treatment (HIGH, contained multiple, animal protein sources; LOW, contained corn and soybean meal as the main protein sources), and time (days 21, 28, and 59 of age) on jejunal mucosa maltase-specific activity. Values are LSmeans ± SEM, *n* = 7. ^a–d^Means without a common superscript differ; *P <* 0.05.

**Figure 3.**
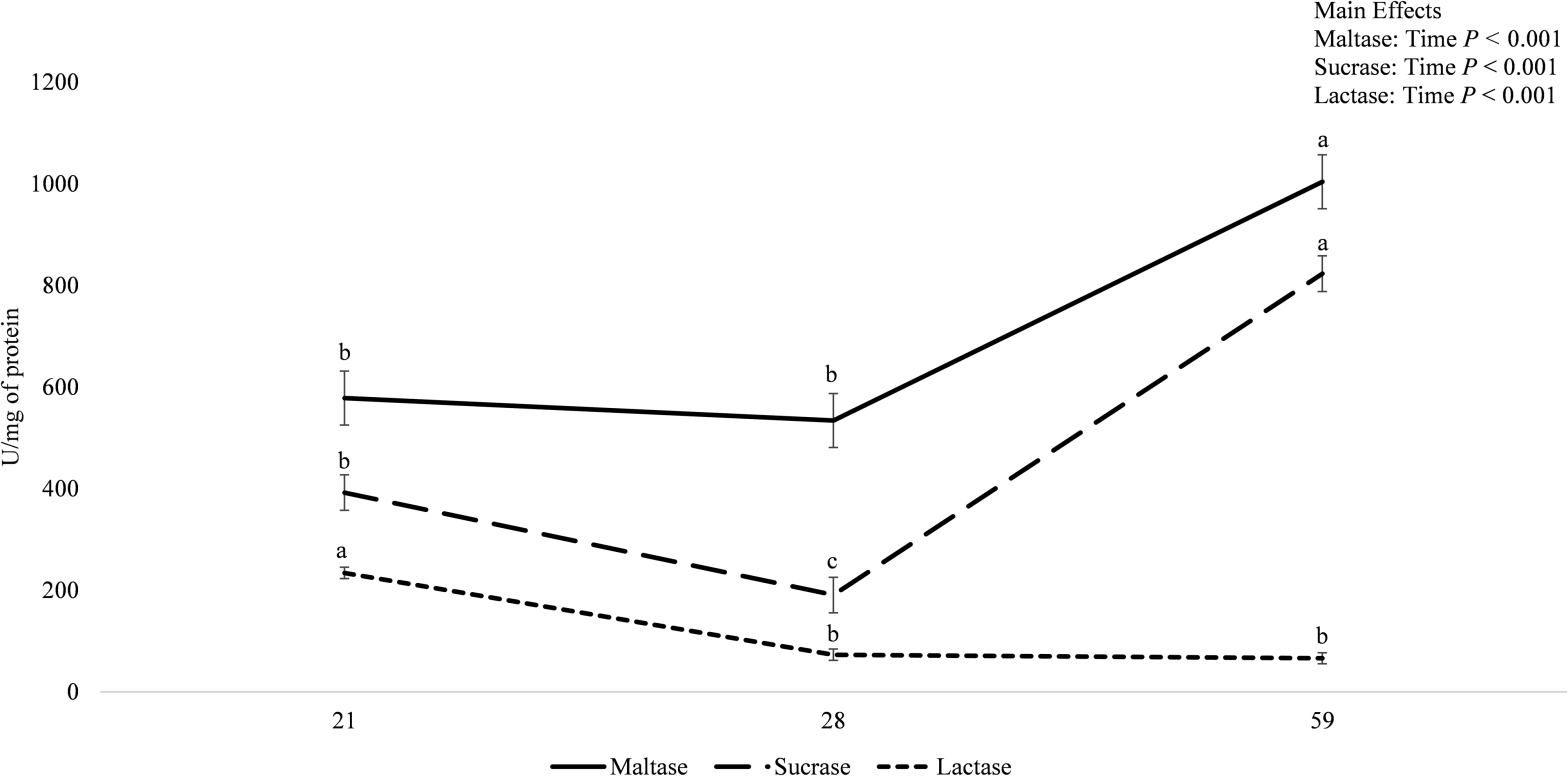
The effect of time (days 21, 28, and 59 of age) on jejunal mucosa maltase-, sucrose-, and lactase-specific activities. Values are LSmeans ± SEM, *n* = 56. ^a–c^Means without a common superscript differ; *P <* 0.05.

The specific activities of jejunal mucosal enzymes were not influenced by the interactions between creep and nursery treatments, or between nursery treatment and time, or by the main effects of creep or nursery treatments (data not shown). Maltase-specific activity was influenced by the interaction between creep feed, nursery treatment, and time (*P* < 0.001; Figure 2). For all dietary treatments except COM-HIGH and COM-LOW, maltase-specific activity was not different between days 21 and 28 of age but greater on day 59 (*P* < 0.05). For pigs that received COM-HIGH, maltase-specific activity was not different between days 21 and 28 but greater on day 59 than on day 28 (*P* < 0.05). For pigs that received COM-LOW, maltase-specific activity was not different between days 21, 28, and 59 of age. On day 59, lower maltase activity was observed for COM-LOW vs. all other treatment combinations (*P* < 0.05) except for pigs that received PMR-HIGH, NO-HIGH, and NO-LOW, which were intermediate. The interaction between creep treatment and time also influenced maltase-specific activity (*P* < 0.05), such that, on day 59 of age, maltase-specific activity was greater than on days 21 and 28 for pigs that received LMR, PMR, or NO (*P* < 0.05; data not shown). Maltase-specific activity was greater on day 59 vs. day 28 for pigs fed COM (*P* < 0.05), while intermediate activity was observed on day 21 (data not shown). Maltase-specific activity was also influenced by the main effect of time (*P* < 0.001), such that, regardless of creep or nursery treatments, maltase-specific activity was greater on day 59 compared with days 21 and 28 (*P <* 0.05), which were not different (Figure 3).

Sucrase- and lactase-specific activities were not influenced by creep or nursery treatments or their interactions (data not shown) but were influenced by the main effect of time (*P* < 0.001; Figure 3). Regardless of creep or nursery treatments, sucrase-specific activity was greater on day 59 vs. day 21 of age (*P* < 0.05), which were both greater than day 28 (*P* < 0.05). Lactase-specific activity was not different between days 28 and 59 which were both less than on day 21 (*P* < 0.05).

## Discussion

The objective of this study was to determine the effect of creep feed composition and form and nursery diet complexity on the evolution of intestinal histomorphology and jejunal mucosa-specific enzyme activities of pigs after weaning. Overall, small intestine histomorphology and mucosa enzyme activities were reduced 1 wk after weaning; however, in most cases, by day 59, histomorphology and enzyme activities recovered or exceeded those determined at weaning. One week after weaning, pigs that provided a low-complexity nursery diet experienced a greater reduction in VH and M in the jejunum than those that provided a high-complexity nursery diet, indicating that the inclusion of highly digestible animal proteins (vs. soybean meal) and greater average daily feed intake in nursery phase I (Christensen and Huber, 2021) reduced the extent of weaning-induced damage to the jejunal villi, as also noted by others (Koo et al., 2017; Ma et al., 2019). Furthermore, the creep feeding regimen did not prevent postweaning villus atrophy and had minimal impact on the evolution of mucosal-specific enzyme activities after weaning.

In the current study, jejunal mucosal maltase-specific activity increased over time, which was expected (McDonald et al., 2010). However, only pigs that received COM during the suckling phase had no difference in maltase activity between days 21 (weaning) and 59, regardless of nursery diet complexity, indicating that maltase activity was already elevated at the time of weaning for pigs fed COM vs. other creep feeding regimens. It is possible that the greater starch content in the COM diet (14% vs. 8% starch for COM and milk-based creep feeds, respectively) promoted jejunal mucosal maltase-specific activity before weaning. A previous study by Byrgesen et al. (2021) found that, at weaning, disaccharidase activity was greater for pigs that provided a pelleted creep feed vs. a liquid feed with matched composition, despite the liquid-fed pigs consuming more feed (on dry matter-basis) for most of the preweaning feeding period. However, in the current study, differences in disaccharidase enzyme activity were not attributed to creep feed form alone. Indeed, in our previous study, LMR pigs had greater dry matter intake during the creep feeding period (Christensen and Huber, 2021), but COM pigs had greater jejunal mucosal maltase-specific activity as demonstrated in the current study.

Similar to maltase, sucrase activity was influenced by time, which corresponds to typical gastrointestinal maturation. Overall, jejunal mucosal sucrase-specific enzyme activity was the greatest on day 59 and lowest on day 28, regardless of creep or nursery feeding regimens, which corresponds to weaning-induced reduction and then adaptation for sucrase activity (Kelly et al., 1991). In addition, lactase-specific activity was greater at weaning than after weaning (days 28 and 59), which was also expected as lactase activity decreases as pigs mature and stop consuming milk (containing lactose as the main carbohydrate; Kelly et al., 1991; Pluske et al., 2003). Others have demonstrated that pigs with greater BW also have greater specific activities of the aforementioned enzymes (de Passillé et al., 1989; Pluske et al., 2003). In the previous study by Christensen and Huber (2021), pigs that received LMR during the suckling phase had greater BW at weaning vs. any other creep treatment but did not have greater jejunal mucosal-specific enzyme activities as demonstrated in the current study. Therefore, heavier pigs at weaning and the provision of additional dietary lactose in milk replacers did not affect lactase-specific enzyme activity at weaning or the progression of maltase and sucrase activities after weaning. Regardless, creep feed composition and form did not have lasting effects on small intestinal histomorphology or specific jejunal mucosal disaccharidase activities after weaning, which corresponds to a lack of creep-feeding effects on growth performance after weaning, despite initial differences in BW at weaning (Christensen and Huber, 2021).

In the previous study by Christensen and Huber (2021), pigs that provided the low-complexity nursery diet had lower average daily gain and average daily feed intake between days 21 (weaning) and 42 of age vs. pigs that received the high-complexity nursery diet, regardless of creep treatment. This corresponds to the greater reduction in jejunal VH and absorptive capacity between days 21 and 28 observed in the current study for pigs fed low- vs. high-complexity nursery diets. Between days 42 and 59, however, no differences were observed for average daily gain or average daily feed intake between nursery treatments (Christensen and Huber, 2021), which aligns with the lack of differences in jejunal and ileal histomorphology and jejunal mucosal-specific enzyme activities by the end of the nursery period (day 59 of age). It should be noted, however, that pigs were randomly assigned to the two nursery treatments based on BW at weaning, but pigs that were assigned to the LOW nursery treatment unintentionally had greater ileal VH on day 21 (at weaning), which may have exacerbated the differences observed in VH during the first week after weaning.

It is likely that higher inclusion of soybean meal combined with reduced feed intake of the low-complexity diets (Christensen and Huber, 2021) was responsible for additional villus atrophy during the first week after weaning. Others have also reported a relationship between increasing soybean meal inclusion (Ma et al., 2019; Koo et al., 2020) and lower feed intake (Pluske et al., 1997) on shorter villi. The maintenance of gastrointestinal morphology after weaning is necessary for pigs to retain the nutrient absorptive capacity and, therefore, feed efficiency (Koo et al., 2017). Brush border enzyme activities (including lactase and sucrase) are greater at the apical end of the villi vs. at the base; therefore, more extensive villus atrophy further diminishes brush border enzyme activities, ultimately reducing feed efficiency (Fan et al., 2001; Tsukahara et al., 2013). Indeed, pigs that received the low-complexity nursery diet had reduced feed efficiency and tended to have reduced apparent total tract digestibility of organic matter on day 28 than those that received the high-complexity diet, irrespective of creep feeding regimen (Christensen and Huber, 2021). Moreover, the stress associated with weaning leads to long fasting intervals and low feed intake resulting in villus atrophy and crypt hyperplasia (Pluske et al., 1997; Boudry et al., 2004; Heo et al., 2018); however, in the previous study, no differences were observed in feeding latency or feed intake during the first 48 h after weaning, regardless of creep or nursery feeding regimen (Christensen and Huber, 2021). However, during the first 7 d after weaning, LMR pigs had a greater average daily feed intake than NO, and pigs fed the high-complexity nursery diet had greater average daily feed intake than pigs fed the low-complexity nursery diet, independent of the creep feeding program (Christensen and Huber, 2021).

In summary, creep feed composition and form did not influence the evolution of small intestinal histomorphology and jejunal-specific mucosa enzyme activities after weaning. Though, exposure to a pelleted creep feed containing 18% starch (as-fed) may increase ileal VH, M, and jejunal mucosal maltase-specific activity at weaning. A low-complexity corn- and soybean meal-based diet exacerbated the transient weaning-induced villus atrophy and reduction in absorptive capacity, but small intestinal histomorphology and jejunal mucosa-specific disaccharidase activities were not different by 59 d of age between pigs that received high- or low-complexity nursery diets. Therefore, high-complexity nursery diets can be used to alleviate physiological modifications to the gastrointestinal tract immediately after weaning but do not offer any long-term benefits in terms of intestinal histomorphology and jejunal mucosal-specific disaccharidase activities. Future studies should examine creep feeding regimens in combination with low-complexity nursery diets when pigs are experiencing an immune challenge or are reared in commercial facilities.

## Abbreviations

CD: crypt depth
CF: correction factor
COM: commercial creep feed
CW: crypt width
HIGH: high-complexity nursery diet with multiple animal protein sources
LMR: liquid milk replacer
LOW: low-complexity nursery diet with multiple animal protein sources
M: absorptive capacity
NO: no creep feed
PMR: pelleted milk replacer
U: units
VH: villus height
VW: villus width
